# Water Pricing Policy as Tool to Induce Efficiency in Water Resources Management

**DOI:** 10.3390/ijerph17103534

**Published:** 2020-05-18

**Authors:** Marcos García-López, Borja Montano, Joaquín Melgarejo

**Affiliations:** University Institute of Water and Environmental Sciences, University of Alicante, 03690 San Vicente del Raspeig, Spain; borja.montano@ua.es (B.M.); jmelgar@ua.es (J.M.)

**Keywords:** water pricing, household budget, household structure, price elasticity, public health, wastewater treatment

## Abstract

This research seeks to determine and analyze the reaction to an increase in the water invoice with the aim of introducing improvements in wastewater treatments. To this end, the purification situation is analyzed and the price elasticities of demand are obtained through Ordinary Minimum Squares. The results show that there are several small noncompliant treatment plants, as well as a complex interaction among consumption, price, and income. In many cases, the results offer small or significant differences without a clear pattern. However, it highlights the number of household members as it is strongly related to consumption and price and not considering it in the elaboration or modification of the water tariff would lead to inefficiencies. A reduction in revenue has also been found due to the strong consumer reaction, so it is necessary to assess in more depth what kind of price change would be most appropriate, as well as whether such measures would be best suited to address the problem.

## 1. Introduction

Water resources are key to all economic activity by accounting for an important part of the economic and social development of the territories [1]. However, they have a significant impact on health and the natural environment if not properly managed [2,3], as scarcity and contamination in water resulting from the activities carried out can generate a strong social, environmental, and economic impact. These aspects adversely affect the quality of life by preventing a reliable supply of water resources [2,4,5], as well as harming the natural environment and public health [6]. Although this impact is greater in developing countries [4,5], the inadequate way in which all wastewater is purified causes advanced countries to also suffer some of the consequences [7].

Currently, water treatment not only reduces discharge pollution but also increases the resources available by generating high-quality water [8]. This implies that treatments must be appropriate so that reuse does not pose a health risk [8,9,10]. It should be noted that a higher degree of contamination has higher treatment costs associated with it, so proper treatment would not only result in significant resource savings but also minimize the impact on health in the long term [9,11,12]. Thus, the proper economic management of water resources allows the elimination of several pollutants that deteriorate the state of the natural environment and can cause diseases in individuals [9,10,13,14,15].

In order to solve the above problems, water policy should assess both the public health of individuals and the state of the natural environment when financing the costs associated with the activity. Thus, the main objective of water pricing is to finance water services, as well as to help induce efficiency and fairness in the system [16]. However, other short-term objectives may result in the total cost recovery not being a priority [16] and, therefore, to the imposition of low prices related to scarcity by promoting excessive water consumption [17]. An example of this is the situation experienced by the Gulf countries, where the low water prices policy is common in a context of great scarcity, so there is no economic disincentive for excessive consumption [18,19]. In these regions, aspects such as legislation, institutional structure, and the need for water for food production are a major barrier to possible price increases [19,20,21]. In the case of Europe, the Water Framework Directive (WFD) is the heart of a regulatory framework on water resource management that gives member states the capacity to price in order to achieve efficient water resource management [22]. In this sense, several countries of the union have a complete cost recovery, even some exceed 100% recovery in terms of operation and maintenance [23]. However, water prices have other effects that should be considered, highlighting effects on demand and water supply. Thus, on the consumption side, higher prices induce more responsible behavior on the part of the consumer, in addition to the relocation of water in the most productive uses [16,24]. On the supply side, the increase in prices leads to an increase in revenue that makes new projects viable, creates incentives to avoid water losses, and allows to improve the management of the service through the improvement of maintenance, employee training, and modern monitoring and management techniques [16].

A relevant aspect in this context is the simultaneous determination of price and consumption, as the price level established influences consumption and this, in turn, will determine the price according to the block of the tariff in which it is located. Therefore, there is a simultaneity bias in the estimates that should be considered when analyzing certain aspects of water pricing [25]. In addition, there may be a bias in the estimates arising from the lack of information received by the consumer [26] because while it is agreed with the late perception of price, whether households respond to the marginal price [25] or the medium, which is easier to perceive [27,28], is a complex issue. As for the consumer’s reaction, a significantly low price of water leads to urban demand being relatively inelastic from price [29] so that price increases slowly increase household spending and its influence on consumption is diminished [30]. However, two important aspects should be considered when measuring the price influence on consumption. On the one hand, lower incomes react more intensely to the price, as the invoice is a relatively higher cost on its budget [31] and, on the other hand, the existence of basic water consumption means that not all consumption is likely to be reduced in the face of price increases [32,33].

In this sense, when setting the water tariff, there are several factors, both demographic and territorial, that can directly influence household consumption. Demographic factors include the structure of the household (number of people, children, age of members, and their likes) as well as the characteristics of the house (year of construction or age of the appliances), factors that are importantly related to consumption and price [34,35]. Specifically, while in smaller households the fixed part of the tariff is a significant proportion of the total, a bigger household distributes this fixed part among more individuals and consumption per person is lower. However, as these households move faster through the sections of the tariff, the variable part has a stronger impact. Differences between household types are, therefore, significant and the inclusion of household typology in pricing policy should be assessed [34,36]. Otherwise, households with more members would be further affected and the policy would not properly meet its objectives, causing unintended effects [37]. In addition, the design of pricing based on the composition and situation of households can bring long-term benefits in those most vulnerable households living in regions with fewer financial resources [5,14,38]. In these cases, a new tariff design could bring benefits and improve the vulnerable situation of lower incomes, either through less impact on their budgets or reinvesting the additional revenue to provide a better service [14,38]. Although this situation is more likely to occur in developing countries, redistribution could also be useful in advanced countries where management, while more advanced, still has room for improvement [5].

As for the regional aspect, several factors can affect water consumption. First, depending on the activity carried out in each territory, water pollution and the cost of purification may vary and affect the natural environment, public health, and quality of life [8,10,14,15,28]. Second, the existence of geographical and/or climatic differences in a territory may explain different levels of consumption, price, and types of water resource management [39]. A key factor in this area is the seasonal period [35], as high temperatures and the arrival of tourists cause an increase in consumption, so the implementation of seasonal tariffs could contribute to efficiency [40]. Thus, even similar regions may differ in price reaction, so the measures implemented must be adapted to the characteristics of the region [41].

For all the above, all influential factors must be considered when making changes to the water tariff. Thus, to modify the tariff structure to avoid the limitations of the standard increasing blocks is possible, for which there are various alternatives. On the one hand, there are alternatives that would not be over-altering the operation of tariffs, such as changing the balance between the fixed and the variable part of the invoice, so that consumer would understand the invoice differently as the cost of consumption increases. In this context, maintaining current prices by implementing measures about the household structure [34,36,42] would allow for greater accuracy in the invoice without penalizing the most vulnerable households. On the other hand, there are alternatives that would further change the operation of the tariff. This would be the case for establishing a home-scale water market where saver households could sell unused assigned water to over-consuming households. In this way, low-income households would save water and financial resources while the most wasteful would pay a penalty, as this extra water endowment would be paid at a higher price than the standard [43]. Finally, charging water based on resource scarcity would also be a major change and would allow the price to inform consumers more precisely about the availability of water resources [42]. However, price measures must be accompanied by regulatory and awareness measures, as well as by knowledge about consumers to have the desired effect [44], in addition to being able to use other forms of demand management [24], that is, price measures should not be considered the only alternatives.

In summary, the determinants of consumption are varied and their interaction is complex. Economic and demographic factors, as well as the climatic, geographical, and physical characteristics of the region, should be considered [45]. On the other hand, differences in the analysis technique used, the level of aggregation available or the characteristics of the data lead to studies showing a diversity of results that complicates a general conclusion [46]. In other words, the general water pricing has been the subject of increasing study but, due to the strong territorial character it presents, a specific study for each situation is necessary. Consequently, to analyze the impact that an increase in the price would have on consumption, public revenue, and household budgets in Spain, differences in rent and residence need to be considered as far as possible.

In this sense, Spain is an interesting case study as it is a country that does not achieve the complete recovery of costs and new measures that generate revenue and/or reduce costs would be suitable [47]. In addition, the tariff in Spain does not consider the characteristics of households (although in specific municipalities being a large family is valued), so its modification could be beneficial. However, since price is determined locally and cost recovery is analyzed by river basin, the analysis should be done considering the differences between territories. The different regions of Spain show differences in terms of climate [48], socioeconomic, geographical, and demographic, which involve the need to disaggregate the analysis as far as possible with the aim of minimizing these differences. However, previous analyses for Spain are scarce and focus on specific territories such as Zaragoza [34], Spanish northwest regions [49], and for certain Spanish cities [50]. These analyses prevent the extrapolation of the results for other Spanish regions that have different characteristics, so this analysis will provide useful information that allows having a more complete perspective about the tariff or other taxes, being able to study its modification to improve wastewater treatments or other water services. In particular, this analysis will allow to know the feasibility of an increase in the tariff in each of the Spanish territories as well as to assess the possibility of modifying its structure because, given the lack of cost recovery, there is a need to make some changes to improve funding. For this purpose, this introduction will be followed by the data used, the methodology applied, the results obtained, the discussion, and the conclusions.

## 2. Materials and Methods

In order to analyze the current situation of wastewater treatment and the possibility of introducing a modification in the water tariff, data about wastewater treatment and the budgetary situation of household in relation to the water invoice is employed. These data allow analyzing the consequences of modifying the water invoice through descriptive evidence, grouping techniques, and econometric regression techniques.

### 2.1. Materials

The analysis of the tariff is based on the data from the *Family Budget Survey* carried out by the National Statistical Institute of Spain (INE). The information used corresponds to the years 2016, 2017, and 2018, controlling by year so that a greater number of observations are obtained, that is, they are used as cross-sectional data. This survey aims to provide detailed information on the budgetary situation of households so that water information is scarce and it is not possible to include certain household characteristics, the price delay, or to correct simultaneity bias. Moreover, the analysis of the purification will be based on basic data on the wastewater treatment plants of one of the regions of Spain, the Region of Valencia, obtained from the website of the Public Wastewater Sanitation Company (EPSAR).

In order to provide a more complete analysis of the modification of the current water tariff, the main variables of interest will be the water consumption in households (cubic meters) and its price (euros), both of annual nature and included in the models in a logarithmic scale. Differentiation between regions and income sections is also critical, so they are used as a filter for estimates of the region of residence and income. In addition, other variables of interest such as the household type, the number of members residing in it, the population density, and the size of the municipality are treated. These last 2 variables are included in all estimates as categorical variables, leaving out dispersed population in the case of density and population less than 10,000 inhabitants in the case of the size of the municipality. It should be noted that the regions of Ceuta and Melilla are excluded as there are not enough observations to filter by income. In addition, to eliminate atypical responses by respondents, values have been removed based on several criteria. In terms of consumption, cases where consumption exceeds 1500 cubic meters per person per year or shows a logarithm of water consumption below −1 have been eliminated. Regarding the price, observations with a unit price of more than 6 Euros or a total invoice higher than 1000 Euros per person are eliminated. Observations with an income that excess 15,000 Euros per month or show a proportion of the water invoice on income above 25% are also deleted. These are very specific situations that occur in very few cases, which is confirmed by the reduced loss of 136 observations out of a total of 61,656. Finally, when dealing with a survey the dataset is only a small sample of total households, so each of these is related to a weighting factor based on how many households it represents.

On the purification side, the flow of treated water, project flow, installed power and the population served are included, as well as yields for the removal of suspended solids (SS), biological oxygen demand (BOD), chemical oxygen demand (COD), and the presence or not of tertiary treatment. Nine observations containing absent data have been removed, in addition to the largest treatment plant in the region, which, with a serving population of 852,799 equivalent inhabitants, is significantly distinguished from the rest.

### 2.2. Methods

The results will be shown in two parts, starting with the wastewater treatment from Valencia and ending with the economic analysis at the national level. For the first aspect, a descriptive evidence table, a cluster model, and a small check of regulatory compliance by treatment plants are included. In this sense, it is checked how much of the purified water remains properly untreated. Detailing the cluster analysis, all variables in the previous section are included to perform the groupings. The number of clusters has been set to 5 so that groups with a too low number of plants are not obtained, the case grouping method is K means, and the association measure is the Euclidean distance.

Moreover, the ordinary least squares technique will be used for economic analysis, which will provide the elasticity necessary to calculate the consumer’s reaction. Unfortunately, the information available is insufficient to correct the simultaneity bias discussed in the introduction, so this technique is used. Once the elasticity has been obtained, it will be used to calculate the consumer’s reaction, which allows finishing the results with the situation after the change of price and the reaction of the consumer.

In the case of Ordinary Least Squares estimates, a total of 136 estimates were made with the aim of comparing them with some depth between the different groups. In particular, 4 sections of income and 17 regions have been used (as mentioned above, Ceuta and Melilla are excluded) so that for each region 8 estimates are obtained, 4 analyzing water consumption and 4 analyzing prices. These estimations allow to deepen the characteristics of households, highlighting, in this case, the income and the 4 sections used, which are: (1) income lower than 1000 Euros per month, (2) income between 1000 and 1999 Euros per month, (3) income between 2000 and 2999 Euros per month and (4) income greater than 3000 Euros per month. The equations are as follows:

(1) $$C_{h}=X_{h}\beta +\varepsilon_{h}$$

(2) $$P_{h}=X_{h}\beta +\varepsilon_{h}$$

where *C* represents water consumption, *P* is the unit price of water, *X* is a vector of individual explanatory variables plus a constant term, *β* is a vector of parameters, and *ε* is a random error term. Subscript *h* refers to the unit of analysis used, households. Therefore, estimating both equations will allow to obtain the influence of certain factors on the consumption and the price of water, thus being able to obtain the value of elasticity.

The main objective of the research is to analyze the effects of the increase in the tariff on household consumption as well as on the revenues of water services. The way in which the reaction estimate will be calculated is to use the elasticities obtained in the consumption and price models. In this sense, the unit price coefficient in price models represents the price elasticity of demand while the coefficient of consumption in price models represents the reaction of the unit price to consumption. Therefore, the elasticity obtained corresponds to the average value based on recent consumption and price data. However, this is not a single step because consumption and price are determined together, that is, the price increase will cause a variation in consumption, which will do over the price and this again over consumption so that a process of returning to equilibrium starts. This balance is achieved because most of the values obtained for elasticity are greater than −1 (in any case negative) as well as the reduced reaction to consumption by the unit price. In particular, as an initial stimulus a price increase of 10% of the current tariff will be introduced, keeping everything else constant, which will cause a reduction in consumption calculated by applying the elasticity obtained through equation (1) to the introduced variation. Since the unit price varies according to consumption, its reduction causes a new variation of the unit price calculated by applying the coefficient of consumption obtained by estimating equation (2) to the reduction of consumption.

## 3. Results

The results achieved after analyzing wastewater treatment, the actual situation of water consumption, and the modification of water tariff are discussed below. This section is divided into 2 parts, the analysis of the wastewater treatment and the estimation of the effects of a tariff modification.

### 3.1. Wastewater Treatment and Health

This section analyzes the current situation of wastewater treatment in the Region of Valencia with the objective of determining whether it is an alternative to introduce a tariff or tax change to cover the deficiencies. In this regard, Table 1 shows some basic data about the operation of treatment plants in this region. In general, the treatment plants with tertiary treatment stand out because they have, on average, greater installed capacity and serve a wider population. In terms of disposal yields, plants with secondary treatment are close to 90% while tertiary plants exceed it. However, secondary plants are much more numerous, so the average depends on them to a large extent.

In order to analyze basic operation differences between treatment plants, a clustering technique is carried out and shown through Table 2 and Table 3. The key factor of the differences lies in the size of facilities, highlighting group 3 with 375 small plants with the lowest yields of all. In this line, the number of noncompliant treatment plants has been included regarding the European regulations, which set a minimum of 90% for SS, 70% for BOD, and 75% for COD [51]. Although this aspect has not been included when grouping, noncompliance plants are concentrated in the group with the smallest facilities, except for 4 of the next group in size. This shows that the problem is small-scale but it should not be set aside, as a total of 90 plants do not meet any of the criteria. Therefore, many small plants take less advantage of the available installed capacity and their efficiency is lower than in bigger facilities. Each of these plants serves a population measured in equivalent inhabitants (ei) of, on average, 917.70, a significantly lower number than the rest of the groups. As the size grows disposal yields improve, so the pollution present in each cubic meter of water is lower. However, tertiary treatment, through water reuse, bears its own relationship to pollution and public health, but since the tertiary plants are distributed throughout the 5 groups, their study should be done separately. In any case, only one plant with tertiary treatment does not meet the disposal yields established by European regulations [51].

Finally, Table 4 includes basic information on the operation of the 90 plants that do not meet any of the criteria for disposal yields. The size of these plants is, on average, very small and yields are significantly far from the average of treatment plants, as well as from the limits established in the regulations. Although they are small treatment plants, the total population they serve is 111,745 equivalent inhabitants, making it a significant population whose water is not being adequately treated, with its respective impact on the natural environment and public health.

This wastewater treatment analysis allows evaluating the option of introducing a tariff or tax modification with the aim of correcting inefficiencies. Since the purification, up to secondary treatment, is financed through a regional tax in this region, it is that would have to be modified. However, the consumer, as discussed in the review, is likely to react to the average water price or the total amount of the invoice. This implies that the reaction of the individuals is based on the price once the local tariff and the regional tax, since in this region both are charged on the same invoice. That is, even if a specific part of the invoice is changed, the consumer reacts to the sum of both concepts. The next section will analyze the consumer reaction to a tariff or tax change with the aim of determining its feasibility, a reaction that would have significant consequences. The first possible outcome would be a no reaction from consumers, so better service funding would be achieved at the expense of household budgets. The second would be a stronger reaction to price variation, which could lead to a decrease in revenue but also a reduction in costs and spill pollution. The third would be an equal reaction to price variation, so household revenues and budgets would not be affected but a reduction in consumption and lower costs would be achieved. Finally, the reaction could be less than the price variation, so consumption and costs would fall and revenues would grow at the expense of households. In addition, the reaction is estimated for the different regions of Spain with the aim of extending the analysis and being able to draw comparisons between regions.

### 3.2. Economic Analysis

Table 5 contains the results for formulas (1) and (2). These estimates include aspects such as the municipality size, the population density, the number of members in the household, the monthly income, and the region in relation to Aragon, which has the lowest consumption per household in Spain according to the data used. The objective is to specify as far as possible the estimation of the parameter of the price and consumption variables, thus having elasticity with greater precision. As the estimates in Table 5 show, the region of residence is relevant when explaining consumption and price. It should be noted that the specific models for each region and income section do not include categorical variables by region, as it has already been filtered based on this criterion.

Through estimating 68 times each model depending on region and income, the reaction of consumption to the price was obtained and vice versa. Table 6 shows the elasticity obtained in each model, in which we can see that consumption varies irregularly between regions and income sections. This is related to the characteristics of each of the income-based household groups. On the one hand, low-income households are already trying to minimize their consumption in order to save as much as possible. Thus, if the price is increased, the low-income households would react to a lesser extent in comparison to those with higher income and consumption, increasing the invoice with low consumption savings and penalizing their budgetary situation. On the other hand, as income rises, consumption tends to be higher by assuming a smaller portion of the household budget. However, they also have a larger savings margin, which allows a stronger reaction in terms of consumption. Therefore, there is a complex interaction between consumption, income, and price since the price has a strong impact on the household budget when income is lower. This leads to savings and a lower margin of reaction to an increase in prices, leading to the current situation with elasticities scattered between regions and income sections.

Once the elasticities are obtained, these are the tool to estimate the reaction of consumers to the variation in price. Table 7, Table 8, Table 9 and Table 10 show the main results, while the tables in the Appendix A show the consumption, price, and weight of the invoice on income both before and after the variation introduced for the different categories. First, Table 7 contains the different representative variables of the obtained result. In general, the final variation in consumption is balanced with that of the unit price, leaving relatively similar the total household invoice as well as the weight it has on the household budget. Thus, revenues vary in a reduced way while consumption is significantly reduced. However, Table 5 already showed significant differences between regions of Spain, so deepening this aspect is essential.

In this sense, large differences can be found between the different regions, which is reasonable since consumption, price, income, and other relevant aspects related to sociodemographic, geographical, and economic characteristics vary significantly between regions. In this case, consumption reacts differently depending on the region analyzed, so a deeper analysis should be done highlighting the effect of the characteristics of each region. As a result, the unit price, although it rises in all regions, also does irregularly, which with the variation in consumption leads to significant differences in the total change of the invoice. In particular, the change in the invoice is distributed between the decrease of 13.72 Euros of the Canary Islands and the increase of 3.71 Euros of Galicia. Nevertheless, in most regions of Spain, the strong reaction to consumption would exceed in proportion to the final increase in prices, and, as a result, the total amount of the invoice would fall.

The consumption and the current price in each case are very important when analyzing the reaction of consumers, which are shown in Table A1. Thus, a household with reduced consumption in search of economic savings or by awareness has a lower capacity to expand savings than a household with high consumption. This significantly conditions the results obtained since not only the reaction is diverse but also this is the situation prior to the price increase. In this way, factors such as income, consumption, and price prior to the stimulus are fundamental, in addition to socioeconomic or territorial characteristics, so more information is needed to be able to analyze this aspect more deeply. For example, Table 8 shows that Canary Islands, Basque Country, and La Rioja present the strongest reaction but, while the first one has relatively high consumption and price and an increase in this would have consequences on the household budget, the other two do not have consumption or a high price. Regarding the smallest reactions, Aragon, Extremadura, and Galicia have a reduced margin to save as they already have a relatively low consumption.

A key aspect when studying water consumption is the household type, especially the number of members residing in the home. In this regard, Table 9 and Table 10 show variations by household type and by number of members, respectively. Starting with the household type, the differences do not vary clearly between the different categories, only the greater reduction in the invoice as the number of members grows highlights. Looking at the results depending on the household members, clearer differences can be detected. In particular, a further reduction in consumption and the invoice as the number of members increases, except for a 16-member household, which is unique in the sample and presents the lowest reaction to consumption and even an increase in the invoice. The households of more than 5 members are not the most frequent, but the important differences found based on this feature mean that not considering it can lead to inefficiencies. Specifically, if the tariff does not consider the number of members, the most populous households will progress fast through the consumption sections, which will make their invoice grow faster. Of course, they also divide the fixed payment of consumption among more individuals, in addition to have more saving capacity, but among those members, there will be a significant number of dependent children without income. However, these households tend to have a higher income than less populated households, so the situation is complex.

In summary, elasticity has been calculated based on the region of residence and household income so that the reaction to consumption could be estimated. The results obtained show differences between the categories analyzed, although in many cases the pattern is unclear. However, it shows the relevance of certain aspects in terms of the reaction to consumption.

## 4. Discussion

The results showed that the reaction by households is associated with a reduction in consumption and revenues of approximately 12.55% and 1.54%, respectively. In addition, thanks to the reduction in consumption and taking as a reference the 100 liters per person per day established by the World Health Organization [52] as adequate consumption, the number of households that exceeds it decreases from 60.29% to 51.88%. In any case, this process would involve a significant number of months due to the delayed perception of the price. Specifying in the Region of Valencia, the result obtained is a decrease in revenues. That is, the reaction to the price would be strong and consumption would fall significantly, so this alternative as a method to improve financing is not adequate. However, this policy would have several benefits that should be considered when assessing its implementation. First, the reduction of consumption allows to minimize the costs of water supply so that the loss of revenues by the city council reduces its importance. However, even in the event of a reduction in costs, the lack of its recovery in Spain prevents the amount of the reduction from being reinvested, as it is necessary to complete the financing. The situation is the same in the case of wastewater treatment because when the consumption diminishes the amount to be treated decreases. Therefore, spill contamination would decrease and pressure on water bodies alleviated. However, these issues should be further explored with the aim of examining whether the loss of revenue would be compensated.

However, this is not the only alternative to be valued with the aim of contributing to cost recovery and water treatments. The results obtained show the importance of considering the structure of the home in pricing, as well as other aspects, so the simple increase of the tariff in its current form would not be efficient. In this situation, other alternatives should be considered, such as altering the balance between the fixed and variable part of the invoice, the inclusion of household types, pricing based on scarcity, or even the water market. The simplest option would be to increase the relative weight of the variable part of the invoice so that the variable cost of the cubic meter would increase. This alternative would require an adequate awareness campaign with the aim of adequately transmitting the impact of consumption on the invoice and, without major price changes, it would be possible to influence consumption. However, if the campaign takes effect and consumption is reduced, it would be necessary to increase price to maintain revenues. In any case, the consumption reduction would contribute to the good state of the natural environment and to public health. Even by altering this balance, implementing the household structure in the tariff would reduce imbalances in their influence on households. In this sense, municipal data could be used to assess the number of members of a household. With this data, the price could be designed to apply specific consumption sections to households based on their members, thus avoiding penalizing the most populated households. In addition, by combining the household type with the relative reduction of the fixed part, single-member households could be prevented from paying a relatively high price derived from the high importance of the fixed part.

On the other hand, alternatives such as pricing based on scarcity or the implementation of a water market would involve structural changes. This would have significant management costs in comparison with those of the other alternatives. The assessment of scarcity would be complicated because more than one municipality is supplied from the same water source. However, it would be a good way to transmit information to the user about the state of water resources. Regarding the water market, although it has certain advantages, it is a complex mechanic that is not very common in Spain.

However, before considering implementing a new pricing policy, it is necessary to consider that there are several factors that may limit its effectiveness. In particular, the need for water resources in the agricultural sector, as well as the legislative and institutional situation of a region, can significantly condition the policy to be implemented [53,54,55,56]. Moreover, the political, historical, socioeconomic, and environmental factors that characterize a region also affect the water policy measures to implement, which are not exclusively price measures [56]. As regards the institutional and legislative aspects, the European normative establishes a system compatible with the design of water pricing policies that pursue the complete cost recovery [22]. The fact that in several countries affected by this regulation there is a water pricing capable of covering all the costs exemplifies the situation in Europe [23]. However, this does not mean the disappearance of all difficulties, as the unequal economic influence of the water invoice depending on regions or household types requires careful development of any price measures. In this sense, fully adapting to the conditions established by European legislation would have an associated impact that water service users would have to face [57]. Portugal is an example of this situation according to its necessity to introduce significant changes to its system in order to comply with the established European level [58]. Consequently, and given the results achieved, the situation is complex and needs to be carefully analyzed with the aim of developing policies capable of inducing efficiency in water resource management.

Therefore, while each of these alternatives would require a study on how it would affect consumption, revenues, the natural environment, and public health in advance, they have advantages and disadvantages. Altering the balance of the tariff and implementing the household type are easier alternatives to apply than assessing scarcity or establishing a water market. In addition, any measure must be approved by the public. In short, it is not feasible to increase the invoice directly, but changes must be made modifying the structure. Therefore, the different alternatives available should be studied with the aim of inducing efficiency in the system. To this end, users’ acceptance of the various measures should be considered, as well as the effect they would have on public revenue, household budget, water consumption, natural environment status, and public health.

## 5. Conclusions

This research seeks to estimate the reaction of water consumption to an increase in the price in Spain. This provides relevant information related to the modification of the tariff or regional tax, so it is useful to deal with problems such as inefficiency in some treatment plants or the lack of cost recovery of water services. Due to the available information, the tool used consists of using the elasticity obtained through a series of estimates so that each parameter corresponds to a specific region and income section.

First, the analysis of the wastewater treatment of the Region of Valencia shows how the size is related to the elimination yields. In this sense, the noncompliant plants are the smallest. With the available data, there has been significance of aspects such as municipality size, population density, household members, household income, and consumption, as well as the price faced by consumers. In addition, the behavior of individuals varies depending on income. As for the elasticities obtained, a complex interaction among consumption, income, and price can be observed. This is because the price has a strong impact on the budget of lower-income households, incentivizing savings, but causing a smaller reaction to a price increase.

The results show how, in Spain, the water invoice varies in a reduced way due to the balance between the increase in the price introduced and the reaction of consumers. However, there is also a reduction in supply and treatment costs, as well as spill pollution and pressure on water bodies. Therefore, the full consequences of the modification should be specified, as well as the effectiveness of this measure in resolving the problem. Deepening by region, important differences have been found and reactions can be associated with factors such as consumption or price prior to the stimulus introduced, as well as income. However, a pattern with current data cannot be found, which lacks enough details about the socioeconomic characteristics of households and territorial factors. In relation to the composition of the household, there has been a further reduction in consumption and the invoice as the number of members increases, which significantly conditions the tariff change.

However, there are several limitations that affect this study. First, as each municipality sets its own price and there are large differences in this regard, the estimated increase as a percentage of the current tariff in each city might not be adequate in practice. Despite this, the analysis considers the volume of invoice of each household and provides relevant information for the future. On the other hand, the lack of time series data and enough information to accurately explain both consumption and price conditions the analysis. In addition, the information available on water purification in the Region of Valencia does not include cost data. These limitations imply the need for more accurate future research. However, the necessary data are not currently collected in Spain.

## Figures and Tables

**Table 1:** Means of the Analysis Variables for the total Treatment Plants and separately for those that have or not Tertiary Treatment.

Variable	Total	Without Tertiary	With Tertiary
**Treated Water Flow (m^3^)**	2106.54	1102.49	15,129.77
**Population Served (he)**	10,015.34	4700.85	78,947.32
**Utilized Capacity (%)**	0.56	0.56	0.53
**Installed Power (kW)**	215.68	129.22	1337.00
**Project Flow (m^3^)**	3540.75	1913.54	24,646.68
**SS (%)**	91.85	91.56	95.62
**BOD (%)**	94.87	94.65	97.68
**COD (%)**	90.32	90.09	93.29

Source: Own elaboration from EPSAR data.

**Table 2:** Basic Data from the Cluster Analysis

Cluster	Frequency	Percentage	Accumulated	Tertiary
**1**	48	10.11	10.11	8
**2**	8	1.68	11.79	8
**3**	375	78.95	90.74	5
**4**	12	2.53	93.26	6
**5**	32	6.74	100	7
**Total**	475	100		34

Source: Own elaboration from EPSAR data.

**Table 3:** Means of Cluster Analysis Variables

Variable/Cluster	1	2	3	4	5
**Treated Water Flow (m^3^)**	2209.25	44,463.75	234.82	17,713.58	7444.84
**Population Served (ei)**	10,698.65	221,658.25	917.70	88,205.83	33,371.13
**Utilized Capacity (%)**	0.61	0.69	0.55	0.61	0.56
**Installed Power (kW)**	373.52	2100.63	37.78	1951.25	941.59
**Project Flow (m^3^)**	4335.29	62,890.00	430.36	30,365.00	13,902.38
**SS (%)**	94.65	95.75	90.90	96.00	96.31
**BOD (%)**	96.27	97.75	94.41	97.00	96.56
**COD (%)**	92.23	93.38	89.69	93.08	93.06
SS excess *	4	0	86	0	0
BOD excess *	0	0	6	0	0
COD excess *	0	0	18	0	0

* Excess variables were not included when grouping. Source: Own elaboration from EPSAR data.

**Table 4:** Averages of analysis variables for those treatment plants that exceed some limit in terms of disposal yields.

Variable	Mean
**Treated Water Flow (m^3^)**	392.71
**Population Served (ei)**	1241.61
**Utilized Capacity (%)**	0.60
**Installed Power (kW)**	43.52
**Project Flow (m^3^)**	680.06
**SS (%)**	78.79
**BOD (%)**	87.12
**COD (%)**	79.78

Source: Own elaboration from EPSAR data.

**Table 5:** Estimation of Models (1) and (2) with National Data.

	Consumption	Price
**Town of between 10,000 and 20,000 Inhabitants**	0.046	0.008
	(0.009) ***	(0.005)
**Town of between 20,000 and 50,000 Inhabitants**	0.041	−0.067
	(0.014) ***	(0.008) ***
**Town of between 50,000 and 100,000 Inhabitants**	0.063	−0.065
	(0.016) ***	(0.009) ***
**Town of more than 100,000**	−0.012	−0.149
	(0.016)	(0.009) ***
**Average Population Density**	0.068	0.008
	(0.009) ***	(0.005)
**High Population Density**	0.097	−0.003
	(0.014) ***	(0.008)
**Number of Members in the Household**	0.104	0.029
	(0.002) ***	(0.001) ***
**Monthly Income of the Household**	0.000	0.000
	(0.000) ***	(0.000) ***
**Consumption Per Household**	-	−0.282
	-	(0.005) ***
Consumption*Income	-	−0.000
	-	(0.000) ***
**Unit Price**	−0.945	-
	(0.013) ***	-
Unit price*Monthly Income	0.000	-
	(0.000)	-
**Andalusia**	0.696	0.341
	(0.014) ***	(0.008) ***
**Asturias**	0.540	0.194
	(0.016) ***	(0.010) ***
**Balearic Islands**	1.047	0.451
	(0.020) ***	(0.012) ***
**Canary Islands**	0.832	0.419
	(0.018) ***	(0.009) ***
**Cantabria**	0.523	0.223
	(0.015) ***	(0.008) ***
**Castilla and León**	0.182	0.031
	(0.017) ***	(0.010) ***
**Castilla-La Mancha**	0.377	0.183
	(0.017) ***	(0.009) ***
**Catalonia**	0.811	0.491
	(0.014) ***	(0.008) ***
**Valencia**	0.787	0.474
	(0.014) ***	(0.008) ***
**Extremadura**	0.441	0.267
	(0.017) ***	(0.009) ***
**Galicia**	0.409	0.160
	(0.016) ***	(0.009) ***
**Madrid**	0.957	0.492
	(0.015) ***	(0.008) ***
**Murcia**	0.982	0.446
	(0.015) ***	(0.009) ***
**Navarra**	0.522	0.276
	(0.017) ***	(0.009) ***
**Basque Country**	0.384	0.132
	(0.014) ***	(0.007) ***
**La Rioja**	0.387	0.110
	(0.018) ***	(0.010) ***
**Year 2017**	0.022	–0.003
	(0.006) ***	(0.003)
**Year 2018**	−0.006	−0.001
	(0.006)	(0.004)
**Constant**	3.814	1.190
	(0.020) ***	(0.022) ***
***R*^2^**	0.39	0.39
***N***	61,520	61,520

* *p* < 0.1; ** *p* < 0.05; *** *p* < 0.01. Source: Own elaboration from INE data.

**Table 6:** Elasticities Obtained by Income Section and Region from Models (1) and (2)

Income Section	1		2		3		4	
Region	C	P	C	P	C	P	C	P
**Andalusia**	−1.35	−0.13	−1.23	−0.15	−1.18	−0.16	−0.71	−0.20
**Aragon**	−0.89	−0.15	−0.66	−0.30	−0.11	−0.43	−0.88	−0.33
**Asturias**	−0.82	−0.17	−1.13	−0.26	−1.09	−0.25	−1.37	−0.23
**Balearic Islands**	−0.45	−0.20	−1.17	−0.06	−1.29	−0.12	−1.25	−0.12
**Canary Islands**	−1.44	−0.14	−1.28	−0.04	−1.82	−0.05	−1.35	−0.27
**Cantabria**	−0.27	−0.16	−1.03	−0.24	−0.88	−0.16	−1.23	−0.10
**Castilla and León**	−0.89	−0.25	−1.05	−0.22	−1.16	−0.16	−1.06	−0.22
**Castilla-La Mancha**	−0.73	−0.21	−0.70	−0.20	−1.12	−0.24	−0.70	−0.20
**Catalonia**	−1.08	−0.37	−0.98	−0.25	−0.86	−0.27	−0.97	−0.30
**Valencia**	−0.99	−0.19	−1.01	−0.20	−0.90	−0.09	−0.91	−0.26
**Extremadura**	−0.36	−0.09	−0.89	−0.11	−0.82	−0.19	−0.73	−0.15
**Galicia**	−0.26	−0.15	−0.55	−0.19	−1.17	−0.15	−0.53	−0.22
**Madrid**	−0.74	−0.01	−0.92	−0.13	−0.82	−0.08	−0.98	−0.33
**Murcia**	−0.67	−0.12	−0.99	−0.19	−1.36	−0.13	−1.04	−0.19
**Navarra**	−1.13	−0.05	−0.96	−0.11	−1.39	−0.13	−0.73	−0.22
**Basque Country**	−1.64	−0.09	−1.46	−0.21	−1.50	−0.11	−1.14	−0.23
**La Rioja**	−1.33	−0.15	−1.34	−0.21	−1.71	−0.16	−0.98	−0.22

Source: Own elaboration from INE data.

**Table 7:** Summary for Spain.

**Consumption Reaction**	**Unit Price Reaction**	**Price Per Person Reaction**	**Invoice Variation**
−12.34	12.66	−1.27	−2.24
**Consumption Per Household**	**New Consumption Per Household**	**Consumption Per Person**	**New Consumption Per Person**
132.76	116.25	64.50	56.51
**Price Per Household**	**New Price Per Household**	**Weight**	**New Weight**
170.62	168.38	0.96	0.95
**Price Per Person**	**New price per person**	**Unit Price**	**New Unit Price**
82.46	81.39	1.44	1.63

Source: Own elaboration from INE data.

**Table 8:** Variations by Region

Region	Consumption Reaction	Unit Price Reaction	Total and Person Price Reaction	Invoice Variation
**Andalusia**	−14.16	12.37	−3.54	−5.50
**Aragon**	−7.33	12.29	4.01	3.41
**Asturias**	−14.59	13.99	−2.67	-3.85
**Balearic Islands**	−12.48	11.45	−2.46	−6.91
**Canary Islands**	−16.68	12.02	−6.70	−13.72
**Cantabria**	−10.62	12.19	0.24	0.09
**Castilla and León**	−13.06	13.10	−1.67	−1.86
**Castilla-La Mancha**	−9.60	12.32	1.52	1.70
**Catalonia**	−13.02	14.35	−0.57	−0.94
**Valencia**	−11.55	12.43	−0.57	−0.92
**Extremadura**	−7.61	11.09	2.63	2.94
**Galicia**	−7.39	11.42	3.16	3.71
**Madrid**	-10.38	12.03	0.37	0.61
**Murcia**	−11.81	12.14	−1.12	−2.88
**Navarra**	−12.21	11.69	−1.96	−2.48
**Basque Country**	−17.66	13.43	−6.60	−7.64
**La Rioja**	−17.81	13.82	−6.47	−7.79

Source: Own elaboration from INE data.

**Table 9:** Variations by Household Type.

Household Type	Consumption Reaction	Unit Price Reaction	Total and Person Price Reaction	Invoice Variation
**An Adult without Children**	−12.23	12.59	−1.23	−1.68
**Two Adults without Children**	−12.33	12.64	−1.27	−2.13
**Other Childless Households**	−12.44	12.70	−1.35	−2.83
**An Adult with at least One Child**	−12.29	12.53	−1.33	−2.18
**Two Adults with One Child**	−12.35	12.66	−1.28	−2.30
**Two Adults with Two Children**	−12.37	12.75	−1.22	−2.48
**Two Adults with Three or More Children**	−12.46	12.80	−1.28	−2.63
**Other Households with Children**	−12.51	12.74	−1.39	−3.19

Source: Own elaboration from INE data.

**Table 10:** Variations by Household Members.

Household Members	Consumption Reaction	Unit Price Reaction	Total and Person Price Reaction	Invoice Variation
**1**	−12.23	12.59	−1.23	−1.68
**2**	−12.32	12.64	−1.27	−2.12
**3**	−12.39	12.66	−1.32	−2.51
**4**	−12.40	12.74	−1.27	−2.60
**5**	−12.51	12.80	−1.34	−3.13
**6**	−12.38	12.77	−1.23	−3.19
**7**	−12.12	12.76	−0.94	−2.88
**8**	−13.08	12.91	−1.88	−5.18
**9**	−14.89	12.57	−4.19	−11.37
**10**	−13.76	12.48	−3.01	−6.76
**11**	−14.98	13.35	−3.62	−9.71
**16**	−9.65	10.92	0.21	0.95

Source: Own elaboration from INE data.

## Data Availability

The Household Budget Survey is available on the Spanish National Statistics Institute website, which can be accessed through this link. As already mentioned during the article, the 2016, 2017, and 2018 editions are used. Finally, it should be noted that the previous web page can be consulted in English, but the design of the survey is only available in Spanish.

## Appendix A

**Table A1:** Values before and after the Price Variation Introduced by Region.

Region	Consumption Per Household	New Consumption Per Household	Consumption Per Person	New Consumption Per Person	Unit Price	New Unit Price
**1**	136.09	117.07	63.88	54.73	1.39	1.56
**2**	78.99	73.26	39.07	36.14	1.17	1.32
**3**	129.87	110.60	72.21	61.86	1.28	1.46
**4**	203.93	178.14	99.40	87.44	1.43	1.59
**5**	154.65	128.79	73.10	61.03	1.46	1.64
**6**	113.21	101.06	58.63	52.72	1.23	1.38
**7**	107.39	93.25	54.97	47.85	1.16	1.31
**8**	109.94	99.20	51.37	46.44	1.24	1.39
**9**	137.04	119.36	66.38	57.58	1.67	1.91
**10**	123.57	109.36	60.46	53.42	1.61	1.81
**11**	101.01	93.05	49.00	45.42	1.31	1.46
**12**	112.96	104.40	56.06	52.25	1.22	1.36
**13**	157.38	140.73	77.19	69.35	1.62	1.82
**14**	164.45	144.53	73.82	65.26	1.43	1.60
**15**	113.64	99.84	56.34	49.50	1.31	1.46
**16**	111.42	92.00	56.01	45.97	1.16	1.31
**17**	122.04	100.33	61.87	50.84	1.16	1.32
	**Price Per Household**	**New Price Per Household**	**Price Per Person**	**New Price Per Person**	**Weight**	**New Weight**
**1**	168.79	163.29	78.66	75.78	1.09	1.04
**2**	83.64	87.05	41.65	43.21	0.45	0.47
**3**	134.55	130.70	75.27	73.48	0.75	0.74
**4**	256.15	249.24	122.84	120.26	1.28	1.27
**5**	204.21	190.48	94.79	88.59	1.42	1.33
**6**	128.76	128.85	66.74	67.23	0.74	0.75
**7**	102.37	100.51	52.35	51.52	0.57	0.56
**8**	121.93	123.63	56.82	57.70	0.76	0.77
**9**	192.67	191.73	92.89	92.24	0.98	0.97
**10**	179.73	178.81	87.54	86.99	1.07	1.07
**11**	123.84	126.78	59.37	61.09	0.82	0.85
**12**	125.58	129.28	62.36	64.65	0.77	0.80
**13**	221.96	222.58	108.96	109.42	1.07	1.08
**14**	214.69	211.81	96.35	95.53	1.30	1.30
**15**	135.05	132.56	66.88	65.55	0.69	0.67
**16**	120.72	113.08	60.69	56.50	0.54	0.50
**17**	122.09	114.30	62.50	58.47	0.68	0.64

Source: Own elaboration from INE data.

**Table A2:** Values before and after the Price Variation Introduced by Household Type.

Household Type	Consumption Per Household	New Consumption Per Household	Consumption Per Person	New Consumption Per Person	Unit Price	New Unit Price
**An Adult without Children**	105.02	92.11	105.02	92.11	1.45	1.63
**Two Adults without Children**	128.27	112.36	64.13	56.18	1.43	1.62
**Other Childless Households**	153.36	133.99	47.49	41.49	1.42	1.60
**An adult with at least One Child**	129.10	113.21	54.68	47.94	1.46	1.65
**Two Adults with One Child**	135.59	118.72	45.20	39.57	1.46	1.64
**Two Adults with Two Children**	150.81	131.99	37.70	33.00	1.45	1.63
**Two Adults with Three or More Children**	164.43	143.87	32.07	28.06	1.45	1.64
**Other Households with Children**	177.55	155.21	38.94	34.04	1.44	1.62
	**Price Per Household**	**New Price Per Household**	**Price Per Person**	**New Price Per Person**	**Weight**	**New Weight**
**An Adult without Children**	133.37	131.69	133.37	131.69	1.17	1.15
**Two Adults without Children**	162.91	160.78	81.46	80.39	0.86	0.85
**Other Childless Households**	196.74	193.91	60.69	59.82	0.85	0.84
**An Adult with at least One Child**	168.52	166.34	71.57	70.64	1.36	1.34
**Two Adults with One Child**	176.70	174.40	58.90	58.13	0.87	0.85
**Two Adults with Two Children**	197.31	194.83	49.33	48.71	0.84	0.83
**Two Adults with Three or More Children**	216.21	213.58	42.15	41.63	1.07	1.06
**Other Households with Children**	230.07	226.88	50.41	49.71	1.02	1.00

Source: Own elaboration from INE data.

**Table A3:** Values before and after the Price Variation Introduced by Household Members.

Household Members	Consumption Per Household	New Consumption Per Household	Consumption Per Person	New Consumption Per Person	Unit Price	New Unit Price
**1**	105.02	92.11	105.02	92.11	1.45	1.63
**2**	127.87	112.02	63.93	56.01	1.44	1.62
**3**	141.96	124.16	47.32	41.39	1.44	1.62
**4**	155.08	135.72	38.77	33.93	1.44	1.62
**5**	175.39	153.28	35.08	30.66	1.44	1.63
**6**	183.65	160.77	30.61	26.79	1.47	1.66
**7**	196.19	171.77	28.03	24.54	1.40	1.58
**8**	200.69	174.71	25.09	21.84	1.41	1.59
**9**	136.82	115.76	15.20	12.86	1.56	1.76
**10**	188.55	162.75	18.85	16.28	1.51	1.69
**11**	364.02	311.59	33.09	28.33	1.32	1.50
**16**	292.20	264.00	18.26	16.50	1.54	1.71
	**Price Per Household**	**New Price Per Household**	**Price Per Person**	**New Price Per Person**	**Weight**	**New Weight**
**1**	133.37	131.69	133.37	131.69	1.17	1.15
**2**	162.67	160.55	81.34	80.28	0.89	0.88
**3**	182.56	180.05	60.85	60.02	0.88	0.86
**4**	202.79	200.19	50.70	50.05	0.86	0.85
**5**	228.79	225.65	45.76	45.13	1.03	1.01
**6**	248.88	245.69	41.48	40.95	1.18	1.16
**7**	251.38	248.50	35.91	35.50	1.22	1.20
**8**	249.26	244.09	31.16	30.51	1.28	1.25
**9**	215.07	203.70	23.90	22.63	1.04	0.99
**10**	252.97	246.21	25.30	24.62	1.17	1.13
**11**	513.55	503.84	46.69	45.80	1.20	1.17
**16**	450.08	451.03	28.13	28.19	1.79	1.79

Source: Own elaboration from INE data.
